# Severe Hindrance of Viral Infection Propagation in Spatially Extended Hosts

**DOI:** 10.1371/journal.pone.0023358

**Published:** 2011-08-23

**Authors:** José A. Capitán, José A. Cuesta, Susanna C. Manrubia, Jacobo Aguirre

**Affiliations:** 1 Departament d'Enginyeria Informàtica i Matemàtiques, Universitat Rovira i Virgili, Tarragona, Spain; 2 Grupo Interdisciplinar de Sistemas Complejos (GISC), Madrid, Spain; 3 Departamento de Matemáticas, Escuela Politécnica Superior, Universidad Carlos III de Madrid, Leganés, Madrid, Spain; 4 Centro de Astrobiología, CSIC-INTA, Torrejón de Ardoz, Madrid, Spain; University of Zaragoza, Spain

## Abstract

The production of large progeny numbers affected by high mutation rates is a ubiquitous strategy of viruses, as it promotes quick adaptation and survival to changing environments. However, this situation often ushers in an arms race between the virus and the host cells. In this paper we investigate in depth a model for the dynamics of a phenotypically heterogeneous population of viruses whose propagation is limited to two-dimensional geometries, and where host cells are able to develop defenses against infection. Our analytical and numerical analyses are developed in close connection to directed percolation models. In fact, we show that making the space explicit in the model, which in turn amounts to reducing viral mobility and hindering the infective ability of the virus, connects our work with similar dynamical models that lie in the universality class of directed percolation. In addition, we use the fact that our model is a multicomponent generalization of the Domany-Kinzel probabilistic cellular automaton to employ several techniques developed in the past in that context, such as the two-site approximation to the extinction transition line. Our aim is to better understand propagation of viral infections with mobility restrictions, e.g., in crops or in plant leaves, in order to inspire new strategies for effective viral control.

## Introduction

Cellular parasites are an ineluctable outcome of the very evolutionary process [1]. Animal and plant cells can be infected by a variety of viruses, usually with a high degree of specificity. The adaptive ability of RNA viruses is a consequence of the vast genetic diversity of their populations, composed by a large number of individuals that replicate their genomes at a mutation rate several orders of magnitude higher than that of cellular DNA [2]. The survival of RNA viruses depends, among others, on host's ability to fight infection, on its capacity to defeat the parasite through the immune system, and on the features of the environment where infection takes place.

To guarantee their survival and propagation, viruses deploy many different and complex strategies that are still poorly known. As a result, our ability to develop specific therapeutic protocols is limited, and the design of control strategies that cause viral extinction represents an important challenge. An often applied method to extinguish viral infectivity is the use of mutagenic drugs that increase the replication error rate of the viral genome. The interference of subpopulations close to extinction or the precise effect of replication inhibitors administered together with mutagens [3] are two other mechanisms under experimental investigation. The effectiveness of increased mutagenesis has been demonstrated *in vitro*, though there is no general agreement on the features of the transition to extinction [4]–[7]. Eigen's quasispecies theory [8] predicted the existence of a maximum value of the mutation rate (the “error threshold”) beyond which the “master sequence”, a particular genome embedded with a selective advantage with respect to any other genomic variant in the population, would disappear from the quasispecies. However, viral extinction through the removal of a master sequence has not been observed in any experimental essay. On the contrary, viral populations capable of surviving only with suboptimal phenotypes have been described [9]. Most likely, strong increases in the mutation rate lead to extinction through mutational meltdown [10]. Extinction may also occur through a progressive decrease in population numbers [5] or as a result of stochastic effects in small populations affected by an increasing production of defective viral forms [11], [12].

There is an on-going effort to introduce more realistic dynamical models able to reproduce empirical results [7], [13], since actual viral behavior often deviates substantially from the predictions of simple models [14]. Classical quasispecies models capture the important fact that a population of genomes replicating at a high mutation rate necessarily has to be heterogeneous at the mutation-selection equilibrium. However, a remarkable drawback of a number of quasispecies models is the assumption that all new mutations have a deleterious effect on fitness, thus neglecting the appearance of neutral and beneficial mutations. This is in plain disagreement with the fact that adaptation occurs frequently. A second (and very often overlooked) key point is the existence of a huge amount of sequences yielding phenotypes that are equally adapted. As a matter of fact, the extreme redundancy of the genotype-phenotype map reveals that postulating the existence of a unique master sequence is not accurate. The inclusion of beneficial and compensatory mutations in models of viral evolution reveals a new class of collective behavior where no error threshold is found, and where extinction occurs through different mechanisms [7].

In addition to intrinsic viral features, such as the replicative ability of a virus and its natural mutation rate, the progress and eventual success of an infection is conditioned by the geometry of the space where it occurs [15], [16]. Infection propagation in cells suspended in a well stirred media or on a lawn, for example, can have dramatically different fates [17]. Experiments with the bacteriophage Q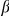 under increased mutagenesis have shown that the number and quality of mutations fixed in populations evolving in liquid medium or in bacterial monolayers is substantially different [18]. These results might give clues to treat viral infections in plants, where leaves, in particular, are well described as two-dimensional tissues. Plant-to-plant propagation in crops is another example with the same geometry. Space induces a remarkably strict clustering of the propagation in crops [19] and even of subpopulations of clonal viruses, a phenomenon observed, among others, in leaves infected with apple latent spherical virus [20]. Mutant phenotypes that remain clustered in a lawn of cells have also been identified in experiments with the bacteriophage T7 [21]. Despite the formation of clusters, high heterogeneity is a property observed in most viruses infecting plants [22]. The relevance of the environment on the evolution of heterogeneous populations has been theoretically studied in several works, paying special attention to the effect of explicit space in the dynamics of the quasispecies [23]. It has been observed that local diffusion lowers the value of the error threshold [24], [25], though it also enhances heterogeneity in the global distribution of types of the quasispecies [17], [26].

In a recent work [27] we reported on a new mechanism of viral extinction due to intraspecific competition for susceptible cells in a space-explicit model of quasispecies. Here, we progress in this line of research and present a novel analysis of the phenomenology associated to populations of viruses propagating in two-dimensional spatially extended hosts. In particular, we describe our results in the light of its biological applications. To this end, we undertake a detailed numerical characterization of the model and measure quantities like the average replicative ability of the virus population, the spatial density of infected cells, the equilibrium distribution of viral types, and the mixing of types that occurs as propagation takes place. Furthermore, we exploit the connection between directed percolation models and the spatial propagation of viruses in situations of restricted mobility. In particular, our model can be regarded as a multicomponent generalization of the Domany-Kinzel (DK) probabilistic cellular automaton [28]. In this work we have used the same numerical and analytical techniques that have been applied in the past to that automaton, such as the two-site approximation to the extinction transition line. This allows us to gain more insight into the features of viral propagation in two dimensions, as well as the mechanisms leading to extinction. In summary, the target and novelty of the present work is to apply diverse theoretical and computational tools to a model of virus propagation, in order to better understand the implications that environments with different geometrical properties might have in devising effective antiviral strategies.

## Methods

In this section we present the model in the context of viral propagation and quasispecies theory, paying special attention to explain the biological observations in which it is based. After describing it in detail, we will consider and analyze the relation between our model and the directed percolation phenomena.

### Model parameters

In our model, each viral particle is phenotypically described by the number 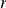 of offspring able to infect healthy cells. Each type in the quasispecies is thus defined through its replicative ability 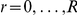. Due to the high error replication rate of RNA viruses, in particular, up to 90% of all virions produced in a single replication cycle might carry lethal mutations or behave as defective particles [29], [30]. As a result, the number of viable and infecting offspring, represented through the quantity 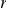, can be orders of magnitude smaller than the actual number of viral particles produced. For instance, in vesicular stomatitis virus only a single viral particle in ten-thousand is able to infect on its own [31].

The microscopic (genotypic) mutation rate affecting the replicating genome translates into a macroscopic (phenotypic) mutation rate that modifies the fitness of the offspring. Though a single mutation may cause an evolutionary advantage, in most instances it produces a mutant with fitness lower than that of the parental virus. Directed mutagenesis in several different viral systems has shown that the ratio between beneficial and deleterious mutations depends on the degree of optimization of the population. Beneficial mutations are about 1000-fold less common than neutral or deleterious mutations in well adapted populations [32], [33], while poorly adapted populations might have a ratio of deleterious versus beneficial mutations up to 10∶1 [34], [35]. Our model does not have a microscopic representation of mutations: it only describes in a phenomenological way its macroscopic (phenotypic) result. We will assume that the offspring of a viral strain can be affected by deleterious mutations (decreasing its progeny production in one unit with probability 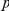) or beneficial mutations (increasing its replicative ability in one unit with probability 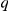). Lethal mutations can hit the class 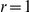 with probability 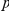. Note that the consideration of a continuous function of changes in fitness applied to the replicative ability under mutation [36] does not modify the qualitative results obtained with this model.

Simple models using the replicator-mutator equation [37] show that, when beneficial mutations are present, phenotypes of high replicative ability can be recovered from mutants of low replicative ability [5], even if mutations accumulate steadily in a genome. Therefore, the inclusion of a non-zero rate 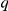 can change drastically the outcome of our model.

A second important feature of our model is the formal implementation of the mechanisms of resistance of the host cells to infection. In plants, most known viral resistance mechanisms target either viral replication or mobility [38]. Mechanisms against replication can be subsumed under parameter 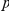, already described. To explicitly represent host defenses other than targeting viral replication, we assume that susceptible cells develop a resistance against infection quantified through probability 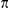, that is to say, a viral particle can infect a susceptible cell with probability 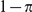.

### Propagation on two-dimensional tissues

**Figure 1:**
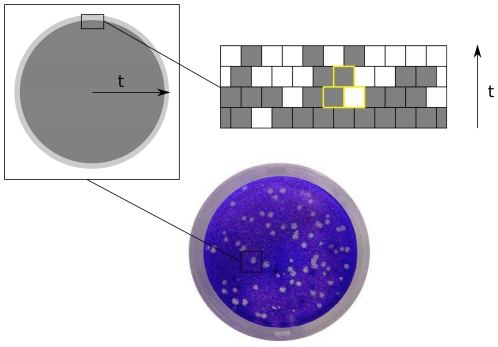
A two-dimensional set-up for viral spreading. In the limit 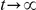 the growth of a lytic plaque in a two-dimensional monolayer is approximated by an array of length 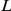 representing the propagation front. Shaded cells are those infected by viral particles. At generation 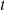, the infective classes occupying two adjacent sites compete with each other to enter a cell and produce an offspring at generation 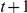 (cells involved are shown with yellow borders).

Lack of susceptible cells can occur in certain spatial configurations or when virus mobility is limited. In [27] we defined a way of infection spreading in two-dimensional environments inspired by an often applied protocol in which spreading occurs on cellular monolayers [39]. The process is started when the offspring of a viral particle infecting a cell is released to the medium after cell lysis. A fraction of that progeny infects adjacent, susceptible cells. The number of infected cells depends on viral mobility limitations. Our model assumes infection to nearest-neighbors, although diffusion to wider ranges of cells can be easily incorporated to the model. This restriction neither represents a loss of generality nor changes qualitatively the results [27]. The process repeats and the size of the (lytic) plaque formed by dead cells grows. After a transient period, all activity occurs at the perimeter of the plaque. If the population does not tend to extinction, the number of cells killed per infective cycle asymptotically reaches a constant value. Even if infection starts off from a single infected cell, it proceeds like the propagation of a front, and this front will become practically flat [17]. Hence we consider a flat propagating front from the beginning. Note that, although the propagation is two-dimensional, the radial growth can be assimilated to time. Therefore cells form arrays in one spatial dimension, and the front propagates perpendicularly and advances one row of cells per generation (see Figure 1).

**Figure 2:**
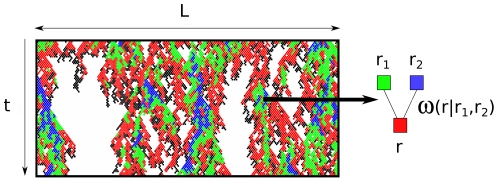
Dynamics of the model of infection propagation close to the extinction threshold. A linear array of infected cells represents the propagation front and produces offspring to infect the next infective cycle. In this example, the maximum replicative ability is 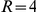. Individuals of each class form clusters (black: 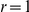, red: 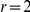, green: 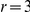, blue: 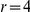). Failure to infect leaves an empty site; success means occupation according to the probabilistic rules given by (2).

Without loss of generality, we assume that the dynamics of the model proceeds in discrete generations on a triangular lattice with periodic boundary conditions (see Figure 2 for a typical configuration close to extinction). Cells are labeled by their position 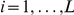 in the row (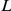 being the length of the array) and the generation 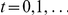 at which the front reaches them.

### Model dynamics

Let 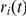 be the replicative ability of the individual occupying site 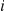 at generation 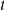. Initially we take 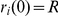 for all 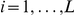. For a given configuration 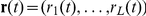 of the array of cells at generation 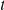, the next configuration can be calculated as follows. Assume that 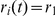 and 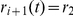. At generation 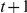 site 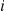 can be infected by one of the 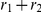 offspring produced by individuals at sites 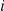 and 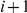, respectively, at the parental generation. Host resistance 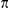 decides whether the cell at site 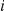 is infected or not. The probability that site 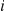 does not become infected after 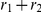 independent trials is 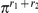. Then infection supervenes with probability 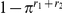. If infection is taking place, the individual that infects 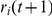 will be with probability 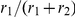 an offspring of the parent with replicative ability 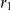, and with probability 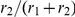 an offspring of the parent with replicative ability 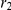. During cell infection the individual is allowed to mutate to neighboring classes. Therefore, the replicative ability of the individual released in the next generation will be
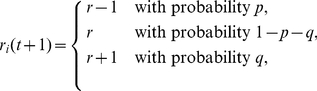
(1)

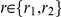
 being the parental replicative ability. Boundary conditions hold for the maximum replicative ability class 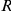. Note that these stochastic rules simply amount to postulating a conditional probability 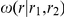 that 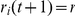, given that the replicative abilities of the individuals attempting to infect are 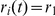 and 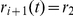, given by
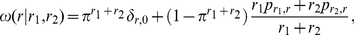
(2)where 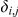 stands for the Kronecker symbol (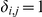 for 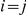 and 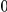 otherwise),
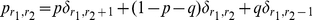
(3)for 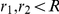 and 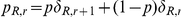. In order to obtain the configuration for all sites of the array we simply apply this local rule to all pairs of sites, using periodic boundary conditions when necessary.

Note that the probability of infection tends to one as the number of viral particles trying to infect a particular cell increases, and tends to zero as the cell resistance improves. Since the entry of more than one viral particle is not allowed, the model implicitly assumes that different genotypes rarely infect the same cell [20]. This is a “winner takes all” rule that could be easily relaxed to account for different processes, including a multiplicity of infection larger than one or the superinfection of a cell.

### Probabilistic cellular automata and directed percolation

The dynamics just introduced describes the global update of an array of cells at each generation. Those rules are in fact a way of defining a (1+1)-dimensional, probabilistic cellular automaton evolving in discrete time to which our model can be easily mapped. In this section, we exploit the mapping between our model and a class of cellular automata to obtain some exact results regarding the transition to extinction of the infection propagation.

Cellular automata are discrete, spatially extended dynamical systems, composed of adjacent cells or sites arranged as a regular 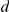-dimensional lattice, which evolve in discrete time steps. Each cell is characterized by an internal state whose value belongs to a finite set. The update is performed simultaneously according to a common local transition rule involving only a neighborhood of each cell. For probabilistic cellular automata, update rules are stochastic. In the discussion that follows, we will only consider (1+1)-dimensional cellular automata.

Among all probabilistic cellular automata, the Domany-Kinzel (DK) automaton is paradigmatic and deserves a brief description. Consider a finite one-dimensional array of 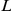 cells. Any of its configurations is determined by an array of stochastic variables 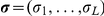 defined at each lattice site at discrete times 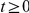. Site 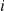 may be in one of the 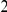 states 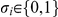 describing active or empty cells, respectively. Note that the array of replicative abilities 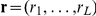 of our model is the counterpart of 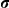, with the difference that 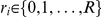. This makes clear in which sense our model generalizes the DK cellular automaton, namely by allowing, at each site, 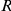 possible active sites instead of one.

Let 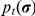 be the probability that state 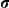 occurs at time 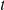. Since any probabilistic cellular automaton is a discrete-time finite Markov chain –i.e., a stochastic process evolving in discrete time steps over a finite configuration space for which the probability of any state at time 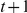 exclusively depends of the state at the present time 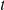
–, the time evolution of 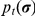 is determined once the (conditional) transition probabilities 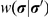 are specified, according to
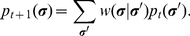
(4)The probability of the transition 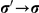 satisfies the conditions 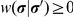 and 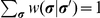. For the DK automaton, transition probabilities are defined as a product of factors associated with each site:
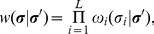
(5)where 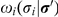 is the conditional probability of finding site 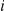 in state 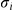 at time 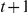, given that the configuration was 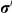 at time 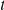. Rules are local, i.e., 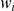 is assumed to depend only on the variables 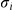 and 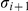 at the previous time step,
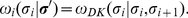
(6)


**Figure 3:**
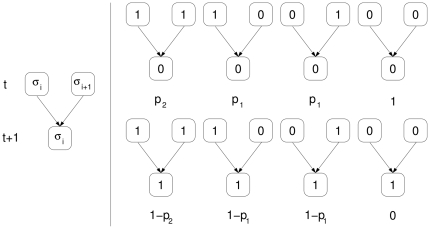
Microscopic transition rules of the DK cellular automaton. Each elementary rule 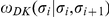 has been specified below each diagram.

Equations (4)–(6), together with the elementary rules 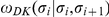 given in Figure 3, define the DK cellular automaton. All transition probabilities are expressed in terms of two parameters 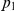 and 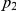. These microscopic rules should be compared with the conditional probabilities (2) of our automaton for viral spreading. Note that the transition 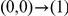 is forbidden, hence the configuration 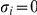 for all 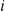 is absorbing, that is, it will persist over time. Something similar occurs with our spatial model. When two adjacent cells are healthy, the probability of infection obviously equals zero (i.e., expression (2) reduces to 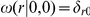). Hence the configuration 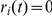 for all 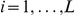 at generation 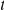 represents extinction of the population and therefore is an absorbing state, as in the DK case. On the other hand, in both models the absorbing state is unique.

For certain combinations of 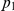 and 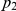, the system evolves towards the absorbing state in finite time. A transition line in the 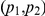 plane separates that phase from an active phase in which fluctuating configurations persist over time (for this phase diagram we refer the reader to [40]). From now on, the extinction regime will be equated to the absorbing state and the survival regime to the active phase.

Numerical simulations confirm that the critical behavior along the whole transition line (except for its upper terminal point 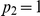) is that of directed percolation (DP). Directed percolation, introduced by [41], was conceived as an anisotropic variant of percolation due to the introduction of a specific direction in space. Such a process models, for example, the propagation of fluids in porous media in the presence of an external gravitational force that singles out a direction of movement of the fluid. Varying the microscopic connectivity of the pores, DP models display a phase transition from a macroscopically permeable to an impermeable state. Originally DP was formulated as a bond percolation (i.e., the connectivity of bonds –pores– determines the set of channels in which the fluid propagates), although the same definition applies for a directed percolation of sites [42]. In fact, it can be shown [40] that the DK automaton contains both types of percolation as particular cases: the choice 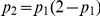 leads to directed bond percolation, whereas directed site percolation corresponds to the choice 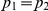.

DP phase transitions occur when the system adopts a configuration in which a connected cluster of active sites covers all the physical space of the system. In both isotropic and directed percolation, the density of active sites serves as an order parameter (i.e., a quantity that is equal to zero in one phase and non-zero in the other), as well as in our model. Numerical estimates for the corresponding critical points of bond and site DP in the limit 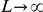 are summarized in Table 1 in the language of the DK cellular automaton.

**Table 1:** Special transition points in the DK cellular automaton [40].

Transition point	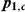	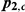
site DP	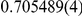	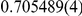
bond DP	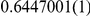	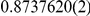

From the point of view of statistical physics, DP models are relevant because of their peculiarities in the transition to extinction. Order parameters are known to exhibit functional dependences near the transition points determined by certain critical exponents. Each set of critical exponents defines a universality class, so that models with different dynamical rules but with the same set of critical exponents exhibit the same universal behavior near the transition. In fact, the DP class covers a wide range of different models, in a way that those models are robust under variations in the microscopic rules. The DP conjecture [43], [44], so far neither proven nor disproven, reflects this fact [40]. It states that a model should belong to the DP universality class if these conditions hold: (i) the model exhibits a continuous transition to a unique absorbing state, (ii) the order parameter that characterizes the transition is positive and scalar, (iii) dynamics involves short-range elementary rules, and (iv) the system has neither special symmetries nor quenched disorder. Although this conjecture has not yet been rigorously proven, it is highly supported by numerical evidence. In particular, the DK verifies the conjecture, which is consistent with the numerical evidence for its belonging to the DP universality class.

In [27] we showed that our model of viral propagation with 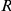 states belongs to the DP universality class. Previously, DP has been also used as a two-state model for epidemic spreading with a transition between survival and extinction of the disease depending on the infection rate [40].

## Results

We start this section briefly reviewing previously obtained results, and rephrasing them in the context of the present article. This is done to serve as an introduction to the features to be described and discussed in the subsequent parts of the section. We will then develop several numerical calculations to generically describe the evolutionary dynamics associated to the model, we will focus in the mechanisms leading to extinction, and will finally develop several approximations to obtain the extinction threshold.

### Summary of previous results

In order to clearly identify the effects induced by propagation on two-dimensional arrays of cells or hosts, it is important to recall the dynamical properties of the infection when propagation occurs in excess of hosts, that is, when physical space is not explicitly considered (e.g. in well stirred liquid media). Most dynamical models of population dynamics are defined in the latter scenario, i.e., they are mean-field models, and it is assumed that resources are abundant enough to allow for an unbounded growth of the population. The detail of the results reviewed in this summary can be found in [17], [27], [45].

There are two important results for mean-field models of the type here discussed that are qualitatively different from the behavior observed in their spatial counterpart. One is the super-exponential growth of the population during the transient prior to attaining mutation-selection equilibrium [45]. Another is the inefficiency of host defenses to counteract the production of sufficiently large amounts of viral progeny [27]. To illustrate these two properties, consider the mean-field version of the model presented previously, where the description is cast in terms of the total number 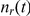 of individuals of class 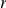 at generation 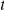:
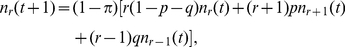
(7)for classes 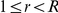, and
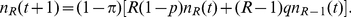
(8)Besides, there is a class 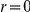 which has lost its replicative ability and is maintained by class 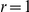 through deleterious mutations, i.e. 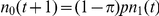.

Consider first the case 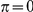 and suppose that, initially, there is a single particle of type 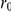 in the population: 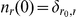, with 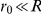, 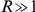. It can be shown that the asymptotic growth of the total population 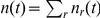 in the regime when 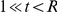 follows
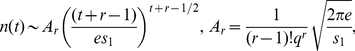
(9)where 
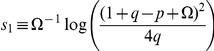
 and 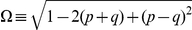. Thus, a population able to increase the number of offspring produced by its individuals through beneficial mutations grows faster than exponentially before the mutation-selection equilibrium is attained [45]. When transients to equilibrium are short, it may be assumed, as most models do, that the dynamics of populations is dominated by their behavior at the mutation-selection equilibrium. However, this does not have to be the case if 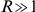: the fast growth of the population could in some cases lead to an exhaustion of the resources before the equilibrium composition can be reached. This situation remains unchanged for other values of 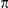, which only affects quantitatively the result above.

Let us discuss now a second situation where 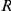 is small enough for the population to attain equilibrium, and consider the case where no beneficial mutations are possible, that is 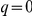. In order for all 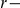
classes to be populated, we take as initial condition 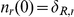. The asymptotic growth rate of the population (at equilibrium now) is 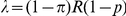, and the fraction of individuals in each replicative class reads
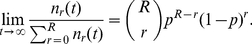
(10)The condition of extinction of the population is obtained in this case from 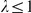, which means that, for a progeny production 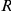 smaller than 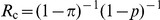, the defenses of the host can defeat the parasite. However, if no restrictions are imposed on the maximum value 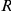, the production of a sufficiently large progeny (just above 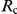) translates into the persistence of the infection. Note that the case 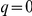 represents the worst situation for the virus. If beneficial mutations are present, the average replicative ability of the population increases, so the virus acquires an additional advantage.

In the light of those two results, it turns out that 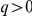 together with an unbounded value of 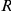 produces an unbeatable parasite, at least if host resistance takes the form of a finite probability 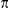 of eluding the infection of each viral particle independently. However, the strategy of increasing progeny production as a mean of beating host defenses fails with the incorporation of physical space [27]. When types compete for the same cell, it turns out that propagation comes to a halt at a finite value of the host's resistance to infection, regardless of the progeny production of the virus. Even in the hypothetical situation 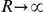, the intraspecific competition for space leads to an effective average value of the replicative ability of the population bounded by the average number of susceptible cells that a viral particle at generation 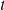 can reach at generation 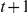. In these conditions, super-exponential growth does not occur, and infection clearance might supervene if the host is able to increase its defenses beyond a finite critical threshold.

### Numerical characterization of the model

The dynamical state of the system can be characterized by the spatial density of active sites (those occupied by particles with 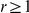) at time 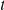,
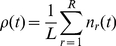
(11)and the average replicative ability of the population at time 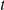 (in the limit 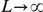)
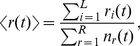
(12)where 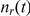 is the number of individuals of type 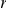 at time 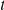. Independently of the initial conditions 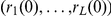 the quasispecies evolves towards an equilibrium distribution where the proportions of all phenotypic classes are maintained constant, and are expressed by
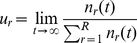
(13)in the limit 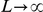.

**Figure 4:**
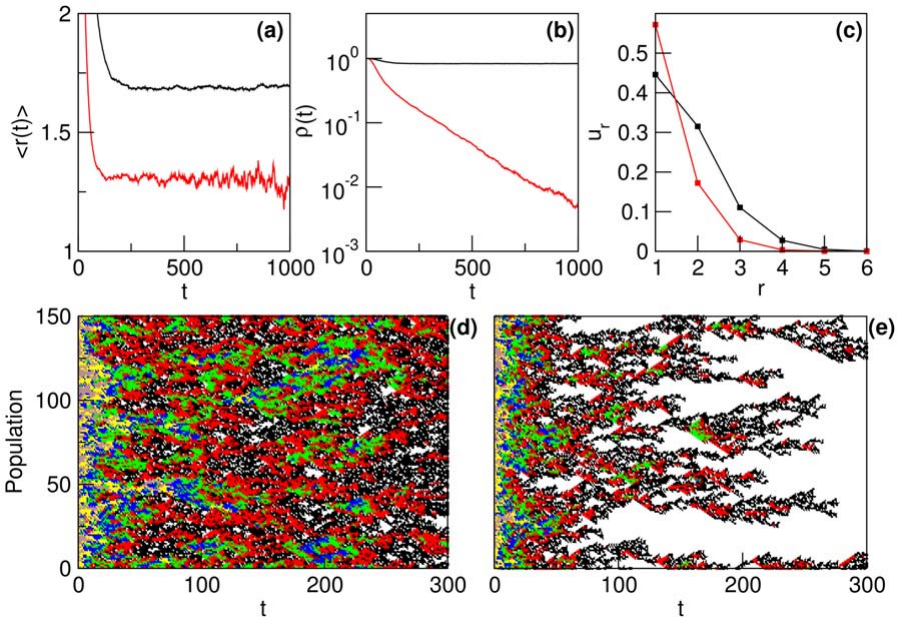
Evolution in time of two populations of 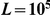 individuals with parameters 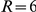, 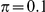, 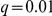, and 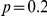 (survival regime, black curves) and 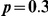 (extinction regime, red curves). The initial conditions are 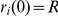 for 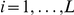. (a) Average replicative ability at time 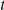, 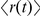. (b) Density of active sites 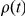. (c) Equilibrium distribution of phenotypes (calculated at 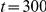). (d) and (e) Detail of the dynamics of the infection propagation in the survival regime and the extinction regime, respectively. Each individual is colored depending on its replicative ability (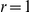, black; 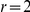, red; 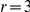, green; 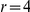, blue; 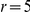 yellow, and 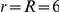, brown). White spots represent individuals of 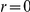 or empty sites.

Figure 4 shows the evolution in time of two different populations, one in the survival regime and the other in the extinction regime. We have paid special attention to (a) the average replicative ability at time 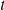 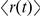, (b) the density of active sites 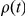, and (c) the equilibrium distribution for 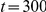. An example of the evolutionary dynamics of both populations is plotted in (d) and (e). Note in Figure 4a and 4c that the tendency towards an equilibrium distribution of phenotypes is also present in the population that gets extinct (although in this case it can only be observed if the time to equilibrium is short enough to reach the equilibrium distribution while the population is still large).

**Figure 5:**
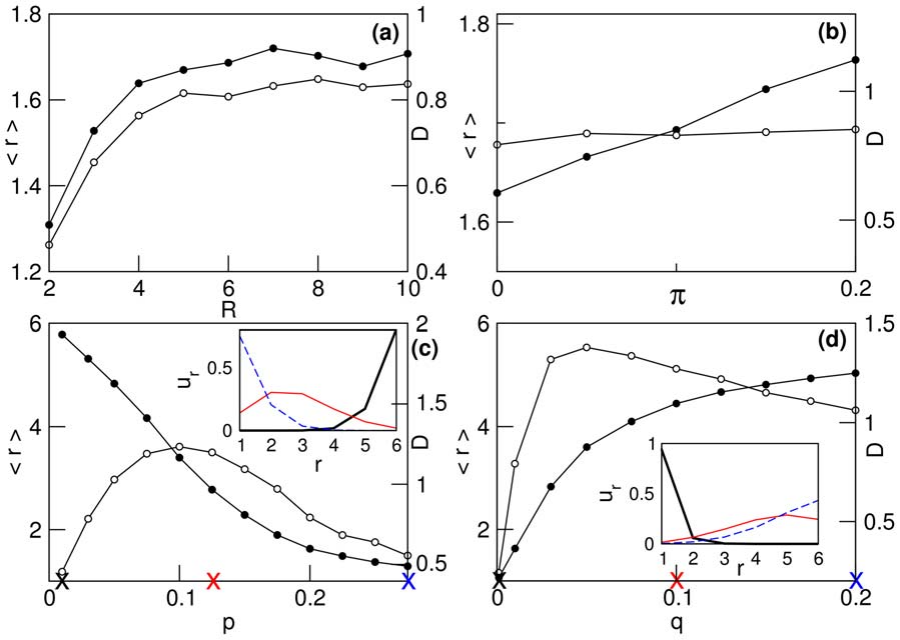
Dependence of the average replicative ability 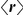 (black circles) and the phenotypic diversity 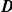 (white circles) at equilibrium on the main parameters of the system: (a) the maximum replicative ability 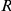 (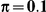, 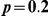 and 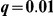), (b) the host resistance 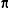 (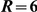, 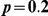 and 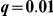), (c) the deleterious mutation rate 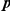 (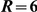, 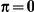 and 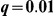), and (d) the beneficial mutation rate 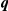 (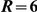, 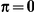 and 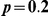). The insets in (c) and (d) show the equilibrium distributions for the values of the parameter marked with crosses (low value of the parameter in black bold, intermediate value in red solid and large value in blue dashed).

Figure 5 shows the dependence of the main properties of the population on the parameters of the model when 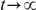, that is, when the population has reached the equilibrium distribution. In particular, we have calculated the average replicative ability 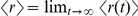 and the phenotypic diversity of the population 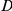 in the equilibrium as a function of: (a) the maximum replicative ability 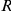, (b) the host resistance 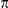, (c) the deleterious mutation rate 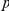, and (d) the beneficial mutation rate 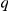. The insets show the equilibrium distributions for the values of the corresponding parameter marked with crosses in the 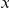-axis (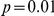 in (c) and 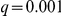 in (d), black bold line), (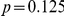 in (c) and 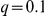 in (d), red solid line), and (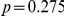 in (c) and 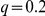 in (d), blue dashed line).

Regarding Figure 5a we see that when the rest of the parameters are kept constant, the average replicative ability 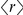 saturates beyond a finite value of 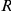. This is at odds with what occurs when there are no spatial constraints. Furthermore, in (b) we observe that 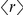 slightly increases with 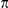, which means that increasing host defenses selects individuals with larger replicative ability. It is precisely the intraspecific competition that causes the saturation of the average replicative ability of the population for increasing 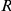, which ultimately leads to the extinction of the pathogen [see the Summary of previous results and [27]]. Finally, in (c) and (d) and the insets therein we can see that 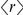 increases with 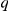 and decreases with 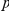, as expected.

As the characteristics of the model do not allow for the existence of multi-peak distributions, the phenotypic diversity of the population in the equilibrium can be measured by its standard deviation,
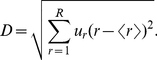
(14)


As it happened with 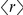, in Figure 5a we see that increasing 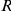 makes the diversity 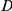 saturate. On the other hand, Figures 5c and 5d show that intermediate values of the mutation rates 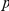 and 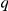 maximize 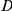 because of a boundary effect imposed by the existence of a maximum replicative ability 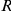 [46], being this effect especially prominent in the equilibrium distributions plotted in the insets.

Furthermore, we have focused our attention in the mixing effect of mutations on the propagating front and their influence in the size of the clusters of individuals with similar replicative ability. This phenomenon has been analyzed studying how the correlation between the replicative abilities of two individuals of the population depends on their distance in the stationary regime. For this purpose, we define 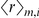, the average replicative ability of individuals at a distance 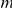 of an individual of class 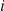, as
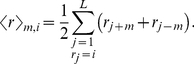
(15)When the parameters allow for the survival of the population and we are not too close to the transition line, this quantity approximately behaves as
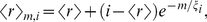
(16)where 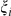 is the typical size of a cluster of type 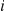. We have numerically observed that 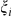 depends on the parameters of the model but is roughly independent of the class 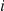 used to calculate it. Hence we can define the typical size of a cluster as 
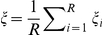
. Regarding (16), we see that 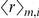 decreases exponentially with the distance 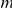 and therefore the memory in this system is short-ranged, which means that the mixing effect of mutations should be very relevant in the destruction of the phenotypic clustering.

**Figure 6:**
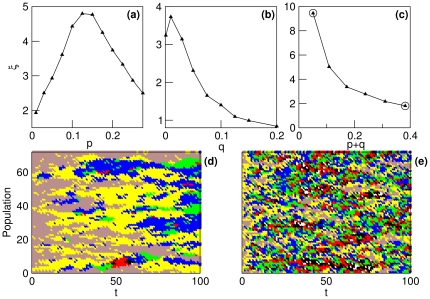
Dependence of the typical size of a cluster 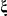 on the mutation rates 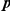 and 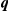 for a population of 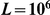 individuals, 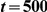, 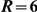, 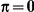, and (a) constant 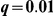, (b) constant 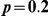, and (c) constant 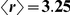. **(d) and (e) Detail of the dynamics of the infection propagation for 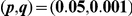 and 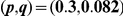 (marked with circles in (c)).** The initial replicative abilities are 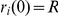 for 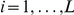. Color code for the replicative ability 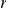 of each individual as in Figure 4.

As we would expect, Figures 6a and 6b show that the typical size of a cluster 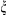 strongly decreases for high values of the mutation rates 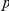 and 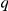. However, note that the multi-parametrical nature of 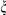 makes that, for low 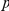 and 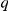 it happens that 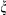 counterintuitively grows with the mutation rates, the reason being that other parameters such as the average replicative ability might influence 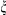 in a non-trivial way (see Figure 5c and 5d for the values of 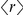 and 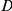 corresponding to Figures 6a and 6b). To overcome this difficulty and in order to study the real effect of 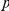 and 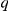 on 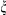, in (c) we have calculated the typical size of a cluster 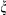 for a wide range of 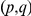 such that the average replicative ability is maintained constant to 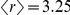. This way we avoid the extremely disturbing consequences of a variable 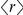 on the equilibrium distribution. The relation between the clustering and the mixing effects of the mutation rates 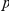 and 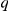 actually becomes monotonic and numerically agrees with 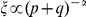, where 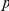 and 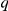 are such that 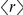 is constant, and 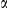 is close to one and slightly depends on 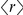. Furthermore, the destructive effect of increasing the mutation rates on the clusters of the propagating front is manifest in Figure 6d and 6e, where we have plotted the dynamics of the infection propagation for the cases marked in (c) with circles, 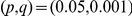 (where 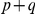 is low, 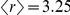, 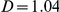 and 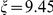) and 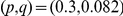 (where 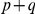 is large, 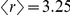, 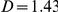 and 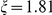), respectively.

**Figure 7:**
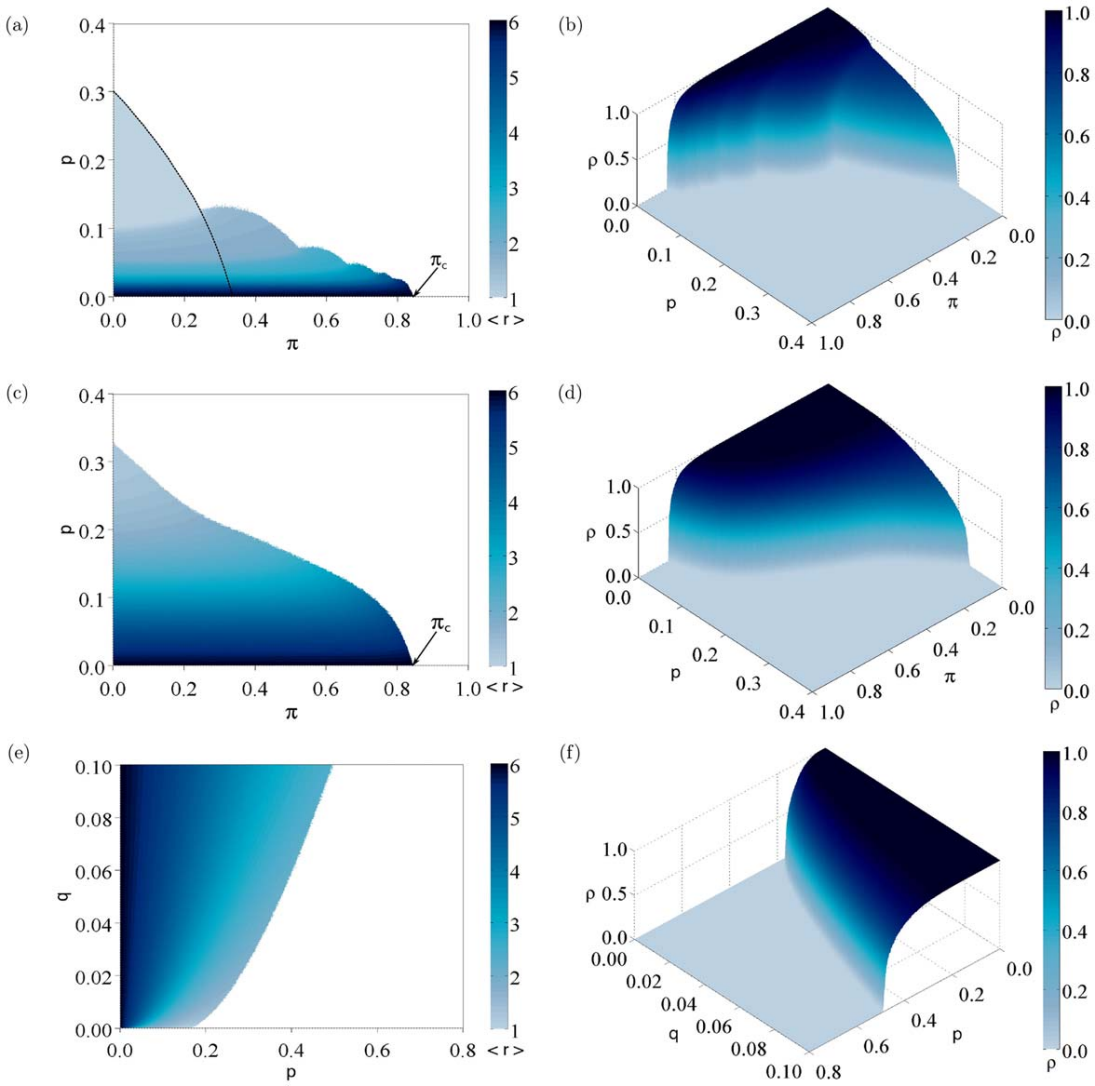
Phase diagrams for 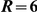 showing the average replicative ability 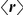 (left plots), and the density of active sites 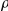 (right plots), as a function of: (a) and (b) 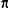 and 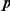 in the absence of beneficial mutations (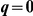); (c) and (d) 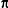 and 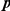 with 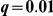; (e) and (f), 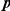 and 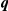 with 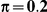. All simulations were developed simulating a system size of 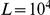 individuals, 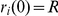 for 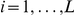, and averaging over 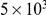 generations after a transient of 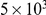 generations.

Finally, Figure 7 shows the average replicative ability 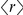 (left plots) and the density of active sites 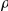 (right plots) when 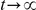 for 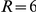 and different values of the parameters 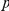, 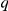, and 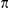. In the phase diagrams that plot 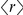, the survival regime is plotted in color and the extinction regime in white, while in (b), (d) and (f) the extinction regime is represented by 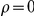. Note that in the absence of beneficial mutations (Figures 7a and 7b) the transition line is in fact the superposition of different transition lines that represent the disappearance of phenotype classes through a cascade of error catastrophes, a phenomenon that is not observed for 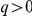. When 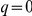, there is no chance to increase the replicative ability of each class, so the behavior of the model is equivalent to that of classical quasispecies models, in which the absence of beneficial mutations leads to error catastrophes [5]. In the present case, the class of higher replicative ability present at a certain time can irreversibly disappear in the same way that the master sequence disappeared from Eigen's model at the error threshold. In our spatial model, the extinction behavior near each error threshold is again that of DP universality class. This can be checked numerically, but in addition there is an exact mapping of the lowest-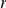 error threshold to a DK cellular automaton. In fact, when only the fitness class 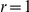 survives, the two non-zero elementary probabilities given by (2) can be mapped to the parameters 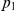 and 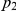 of the DK cellular automaton,
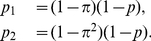
(17)This yields the black line shown in Figure 7a.

The extinction threshold for the highest class 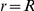 (marked in Figures 7a and c with arrows) can be also calculated thanks to a mapping with a DK automaton in the absence of deleterious mutations. Since the initial condition is formed by individuals of the maximum replicative ability and 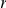 is not allowed to decrease (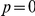), sites can be either occupied or empty. This maps our model to a DK automaton defined by
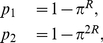
(18)which satisfies 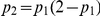 and therefore corresponds to the directed bond percolation case. This yields an upper bound for the value of 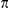 beyond which the propagation will not progress,
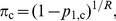
(19)where 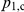 is the critical value of the bond directed percolation case (c.f. Table 1). From this expression it is apparent that 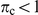 for finite values of 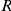. Note that if host defenses are above that threshold at 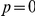, the spatial configuration (or any other situation where viral mobility limitations are relevant) causes the extinction of the pathogen. This fact is different to the situation of excess of available cells (see the summary of previous results): for a certain value 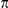 of host defenses, the pathogen can increase its maximum progeny production 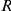 above a critical threshold (equal to 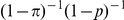 for 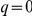, the worst case scenario), which permits the survival of the virus. As a result, this mechanism produces a runaway co-evolution between the virus and the host in situations of excess of resources.

### Approximations to the transition line

To gain more insight into the mechanisms leading to extinction in our spatial model, it is useful to make approximations to the density of active sites and the transition line. Standard techniques have been applied in the past to probabilistic cellular automata [47], [48], in particular, to the DK cellular automaton. This methodology can be extended to our model as well.

Such approximations use the fact that probabilistic cellular automata are discrete-time Markov processes and therefore satisfy a dynamical equation [analogous to (4)] for the probability 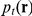 of a given configuration of replicative abilities 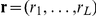 occupying the lattice at time 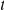. This way, we can write down a hierarchy of equations for the one-, two-, 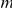-site marginal probabilities. Such a hierarchy is obviously infinite, but can be closed at a certain level using an approximate closure relation between marginal probabilities.

Let 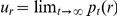 be the asymptotic density of cells occupied by a virus of class 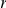, where
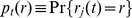
(20)is the marginal probability that a given cell (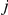) is infected by a viral particle of replicative ability 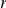 (remember that 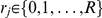). The time evolution of the one-site distribution,
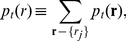
(21)according to (2) and (4), is
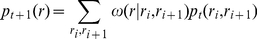
(22)where 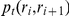 is the marginal probability distribution for two adjacent sites. Further, the two-site marginal probability is coupled to the three-site probability, so
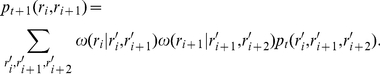
(23)Recursion on these formulae leads to an infinite hierarchy of equations. The 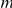-site approximation consists of truncating the hierarchy by estimating the (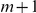)-site probabilities on the basis of those for 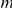 sites.

The one-site approximation factors the two-site marginal probability out as a product of one-site probabilities,
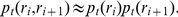
(24)This relation provides a closure of the hierarchy and transforms (22) into the following nonlinear system for the asymptotic densities of each viral class,
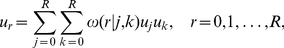
(25)together with the normalization condition 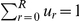.

Two-site approximations take into account two-site marginal probabilities,
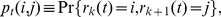
(26)the next in the hierarchy [Eqs. (22) and (23)]. It can be closed at this level by approximating three-site probabilities in terms of two-site quantities, using conditional probabilities:
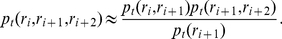
(27)Additionally, one-site probabilities satisfy 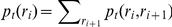. Hence the hierarchy closed by (27) reduces, in the asymptotic limit 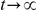, to the non-linear system
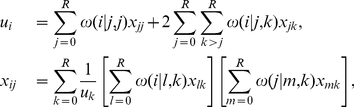
(28)for 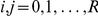, and where 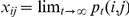 denote two-site correlations. Thanks to the symmetry 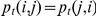, the number of independent correlations reduces to those which satisfy the constraint 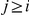. The consistency condition 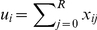 as well as the normalization condition 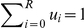 must be satisfied. Approximations involving higher order correlations are too cumbersome for this automaton.

**Figure 8:**
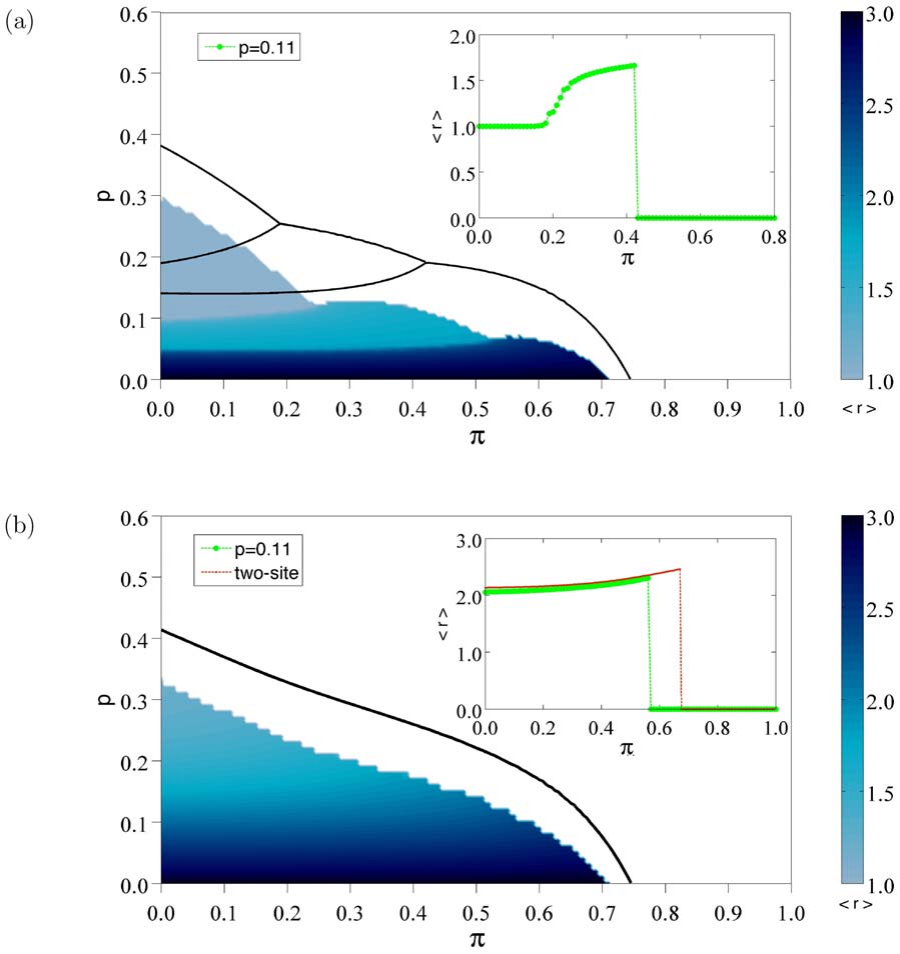
Phase diagrams for 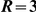. (a) Contour plot of the average replicative ability 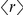 as a function of 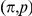 in the absence of beneficial mutations (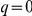). (b) 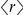 in the 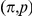 plane when beneficial mutations are considered (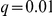). The initial replicative abilities are 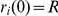 for 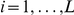. Two-site approximations to the critical thresholds are shown as black curves, whereas simulation results appear in a color scale coding for the average replicative ability 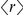. Insets correspond to cross-sections of the surface at 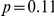 (green curves). For 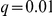 we also show the dependence of 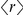 with 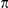 under the two-site approximation regime at fixed 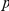 (red curve).

We have checked the accuracy of the two-site approximation for a maximum replicative ability 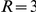, both in the presence (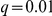) and the absence (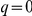) of beneficial mutations. Results for the average replicative ability are summarized in Figure 8. For 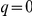, the two-site approximation recovers qualitatively the sequence of error thresholds observed in simulation (the same phenomenon was shown in Figure 7a for 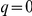 and 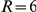). Although the comparison with the transition line is not satisfactory, far away from the transition the surface of 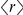 as a function of 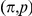 (at constant 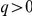) is well predicted, as we observe from the inset of Figure 8b. Therefore, these approximations capture the global dependence of the average replicative ability, although the approximation of the critical threshold is poor.

**Figure 9:**
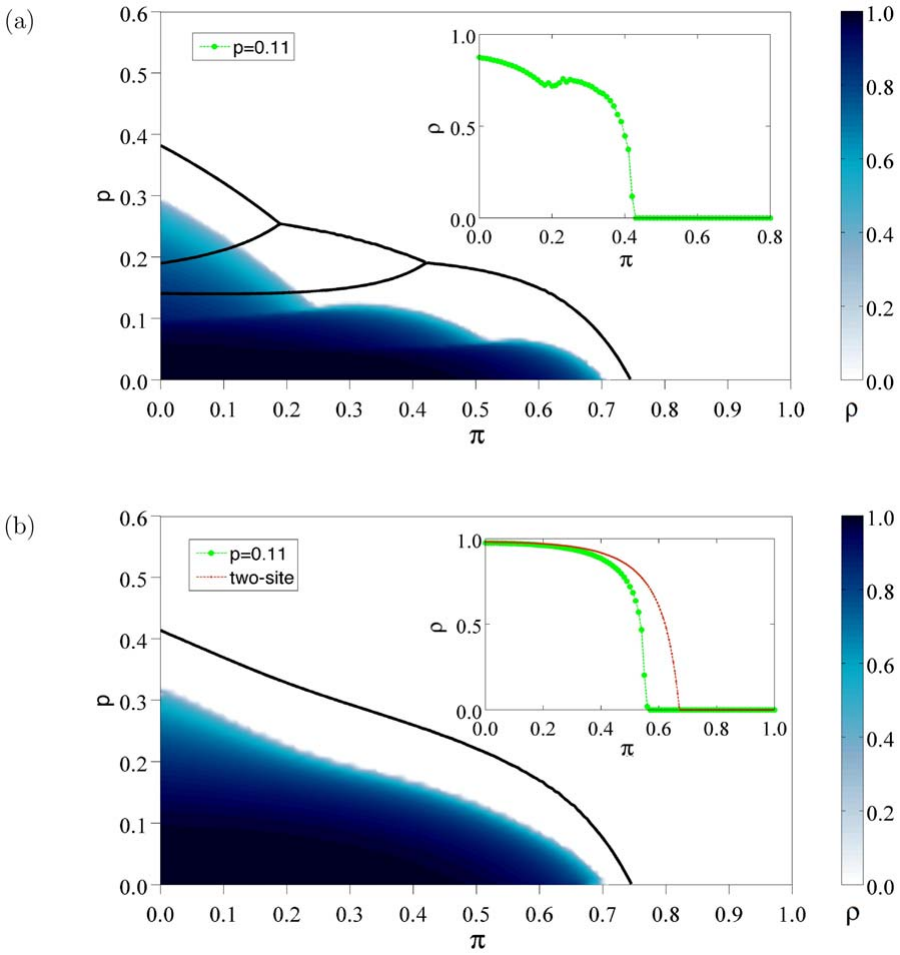
Phase diagrams for 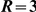. Variation of the density 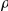 of infective particles for 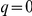 (a) and 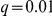 (b). The initial replicative abilities are 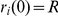 for 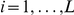. Two-site approximations to the critical thresholds are shown as black curves, whereas simulation results appear in a color scale coding for density of active sites. Insets are cross-sections of the surface at 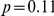 (green curves). For 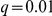 we also show the dependence of 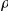 as a function of 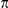 under the two-site approximation scheme at fixed 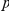 (red curve).

In Figure 9 the density of infected sites (11) is plotted in the 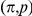 plane. Similarly, the two-site approximation produces accurate results away from the transition, although the prediction of the critical line is only qualitative.

In the Supporting Information S1 we show how this technique can be used to obtain analytical approximations to the transition line in the simplest case of maximum replicative ability 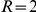.

## Discussion

In this work, we have developed a general description of a two-dimensional model of quasispecies propagation, paying attention to the dependence of the evolutionary dynamics on the probabilities of undergoing a beneficial and a deleterious mutation at rates 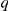 and 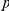, on the maximum replicative ability 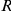, and on the parameter accounting for the host resistance to infection 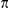. We have analyzed the mixing effect of mutations, which destroys the clusterization of the population as the mutational probabilities increase. Furthermore, all transitions occurring in the spatial environment studied belong to the DP universality class, be they the loss of the highest-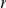 class present when advantageous mutations are absent or the global extinction of the population when beneficial mutations are considered. To the best of our knowledge, this mechanism is different from all other extinction transitions described in models of evolving populations so far.

When there is competition for susceptible cells within the different classes that form the quasispecies, the transition line between the infective (or survival) and the non-infective (or extinction) phases has been calculated by numerical simulation upon variations on the mutation rates 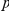 and 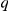 and the host resistance 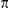. We have performed approximations to the transition line under the one- and two-site approximation regimes. The correct exponents of the DP transition cannot be recovered under this approximation scheme, since it is but a mean-field approximation to the dynamics. This notwithstanding, our approximations lead to fairly good analytical results when the maximum replicative ability 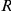 is not too large. As a matter of fact, we have checked that any approximation based on two-site correlations decreases its accuracy as 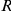 increases. The reason is simple: when multiple viral types of different replicative abilities coexist, the range of the correlations between types becomes wider and an approximation based in just two sites is not enough to reproduce correctly the behavior near the transition line.

The generation of large progeny numbers is usually interpreted as an adaptive strategy of viral populations. In particular, it is broadly accepted that the high mutation rates of RNA viruses are combined with a large progeny production in order to enhance the diversity of the population, thus maximizing the chances of successful infection. Previous studies identified over-production of viral progeny in spatial infections as a by-product of competition within the quasispecies [17]. In a mean-field scenario (e.g. in well stirred liquid media), and taking host resistance to infection into account, offspring over-production appears as an adaptive strategy to overcome host defenses. When the availability of susceptible cells is unlimited (or just not explicitly considered), host defenses are thus unable to counteract a sufficiently large increase in the number of offspring, with the result of a run-away co-evolution between virus and host [27]. However, the explicit consideration of the physical space dramatically changes the situation. First, irrespectively of the maximum number of offspring 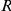 that a virus can produce, there is a finite value of host defenses able to halt its propagation. Second, the super-exponential growth observed during the transient towards mutation-selection equilibrium in the mean-field model disappears. It is the saturation of the average replicative ability of the population to a finite value, due to limited resources, what explains those major changes in the dynamical behavior of the system.

Our model is relatively simple when it comes to the actual mechanisms that plants, for instance, have developed to fight pathogens. However, it could be extended to account for more realistic situations. For example, many plant species present genetic polymorphism for susceptibility to a particular virus [49], i.e., different individuals might have variable degrees of resistance to viral infection and spread. Our model can be extended to account for infection propagation in crops or in tissues formed by non-identical cells by allowing the resistance probability 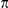 to be host-dependent. The spatial configuration should then be cast as a heterogeneous distribution of individuals occupying fixed positions in space. This would introduce a form of quenched spatial disorder that may lead to universality classes for the extinction transition different from DP [40]. Spatially quenched disorder could change the properties of viral extinction to those of dynamic percolation [50]. The scenario we have studied is a first step towards tackling new situations where different factors like individual variations in host resistance, co-evolution of the relevant parameters, or host superinfection could be made explicitly. Current knowledge of the phenomenology of percolation phenomena in different situations might inspire new strategies to stop viral propagation in different environments.

## Supporting Information

Supporting Information S1Obtention of the analytical approximations to the extinction transition line in the simplest case of maximum replicative ability 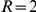 under the one- and two-site approximation schemes discussed in the main text. These approximations qualitatively capture the behavior observed in simulations for the transition to extinction.(PDF)
