# Supplementary material for: Severe Hindrance of Viral Infection Propagation in Spatially Extended Hosts
Source: PLoS One. 2011 Aug 23;6(8):e23358. doi: 10.1371/journal.pone.0023358 (PMC3160299; doi:10.1371/journal.pone.0023358)
Supplement: Supporting Information S1 — Obtention of the analytical approximations to the extinction transition line in the simplest case of maximum replicative ability under the one- and two-site approximation schemes discussed in the main text. These approximations qualitatively capture the behavior observed in simulations for the transition to extinction. (PDF) [file pone.0023358.s001.pdf]

SUPPORTING INFORMATION of Severe hindrance of viral infection propagation in spatially extended hosts

José A. Capitán[1,2], José A. Cuesta[2,3], Susanna C. Manrubia[4], Jacobo Aguirre[4,*]
1 Departament d'Enginyeria Informàtica i Matemàtiques, Universitat Rovira i Virgili, Tarragona, Spain
2 Grupo Interdisciplinar de Sistemas Complejos (GISC)
3 Departamento de Matemáticas, Escuela Politécnica Superior, Universidad Carlos III de Madrid, Leganés, Madrid, Spain
4 Centro de Astrobiología, CSIC-INTA, Torrejón de Ardoz, Madrid, Spain
∗ E-mail: aguirreaj@cab.inta-csic.es

1 Supporting Information S1. Analytical approximations for $R = 2$

The simplest case $R = 2$ of our model allows for analytical solutions of the transition lines in the one- and two-site approximations.

Under the one-site approximation, the densities $\mathbf{u} = (u_1, u_2)$ of infective classes are the roots of the quadratic system

$$\mathbf{a}_j\mathbf{u}^\mathsf{T} - (1-\pi)^2\mathbf{u}\mathsf{M}_j\mathbf{u}^\mathsf{T} = 0, \qquad j = 1, 2, \tag{S1}$$

with vectors

$$\begin{aligned} \mathbf{a}_1 &= (2(1-\pi)(1-p-q) - 1, 2(1-\pi^2)p), \\ \mathbf{a}_2 &= (2(1-\pi)q, 2(1-\pi^2)(1-p) - 1), \end{aligned} \tag{S2}$$

and matrices $\mathsf{M}_{1,2}$ given by

$$\mathsf{M}_1 = \begin{pmatrix} 1-p-q & \frac{1}{3}[(1-q)(2+\pi) - p(1-\pi)] \\ \frac{1}{3}[(1-q)(2+\pi) - p(1-\pi)] & (1+\pi)^2 p \end{pmatrix} \tag{S3}$$

and

$$\mathsf{M}_2 = \begin{pmatrix} q & \frac{1}{3}[q(2+\pi) + (1-p)(1+2\pi)] \\ \frac{1}{3}[q(2+\pi) + (1-p)(1+2\pi)] & (1+\pi)^2(1-p) \end{pmatrix}. \tag{S4}$$

The system (S1) is difficult to solve in general for $q > 0$. However, we can devise heuristic approximations as follows. Near the critical line, the densities $u_{1,2}$ are close to zero, so terms involving u_1^2 and u_2^2 can be neglected. This simplifies the system considerably. The resulting system can be solved and yields the transition line

$$p_{1\mathrm{s}}(\pi, q) = \frac{2 - \pi - 4\pi^2 + \sqrt{\pi^2 + 8q(1 - 3\pi^2 + 2\pi^4)}}{4(1-\pi^2)} \tag{S5}$$

in the one-site level of approximation.

Analytical results can also be obtained at the two-site level of approximation. For $q > 0$ the nonlinear system (28) of the main document is too complicated to be solved analytically. A numerical resolution of that system for $R = 2$ yields the phase diagrams of Figure S1 for $\langle r \rangle$ and ρ. This nonetheless, we can provide a heuristic, analytical approximation to the whole line under the two-site approximation scheme.

Figure S2 shows a typical dependence of two-site correlations as functions of p. In the inset we observe that, close to the critical threshold, $x_{01} \approx x_{11}$ and $x_{02} \approx x_{12} \approx x_{22}$. By imposing that $x_{01} = x_{11}$ and $x_{02} = x_{12} = x_{22}$, the system (28) of the main document reduces to a linear, homogeneous system in

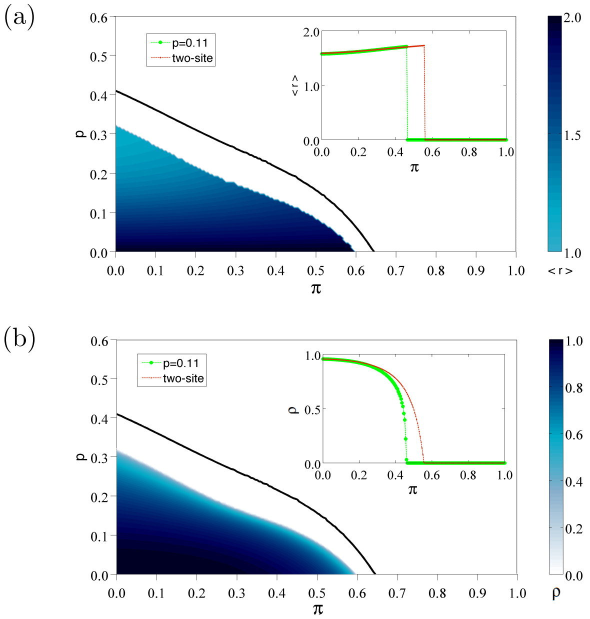

Figure S1. Phase diagrams for $R = 2$ and $q = 0.01$. (a) Average replicative ability $\langle r \rangle$, and (b) density of active sites ρ. Two-site approximations to the critical thresholds are shown in black, whereas simulation results appear in a color scale coding for $\langle r \rangle$ (a) and ρ (b). Insets show the dependence with π at fixed $p = 0.11$ (green curves).

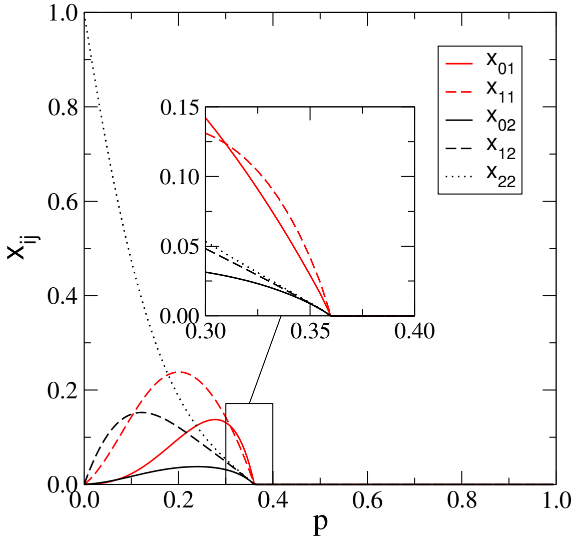

Figure S2. Heuristic approximation to the transition line in the two-site approximation. The dependence, as functions of p, of the two-site correlations for $R = 2$ is depicted. Remaining parameters are $q = 0.02$ and $\pi = 0.15$.

variables (x_{01}, x_{02}). The approximate critical line is obtained equating to zero the determinant of the

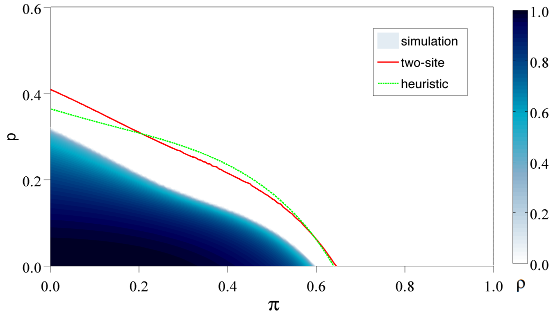

Figure S3. Heuristic approximation to the transition line in the two-site approximation. The analytical curve (S6) is compared with simulation results for $R = 2$ and $q = 0.01$.

system matrix, and takes the form

$$p_{2s}(\pi, q) = \frac{B_1(\pi) + \sqrt{B_2(\pi, q)}}{B_3(\pi)}, \tag{S6}$$

with the polynomials

$$\begin{aligned} B_1(\pi) &= -25 + 9\pi + 50\pi^2 + 42\pi^3 + 26\pi^4 + 6\pi^5, \\ B_2(\pi, q) &= (-1 + 17\pi + 14\pi^2 + 6\pi^3)^2 - 2qB_3(\pi)(-7 - 5\pi + 14\pi^2 + 18\pi^3 + 13\pi^4 + 3\pi^5), \\ B_3(\pi) &= 2(1 - \pi)(3 + \pi)(13 + 13\pi + 7\pi^2 + 3\pi^3). \end{aligned} \tag{S7}$$

We compare in Figure S3 the threshold curve (S6) with simulation results. As we can see, this formula fairly reproduces the transition line at the two-site level.
